# Assessment of a self‐inspection method and reporting measured electrometer correction factors for reference‐class electrometers in radiotherapy

**DOI:** 10.1002/acm2.14082

**Published:** 2023-06-26

**Authors:** Motohiro Kawashima, Maria Varnava, Shuichi Ozawa, Hiroki Okada, Hiromitsu Higuchi, Yoshihiko Hoshino, Mutsumi Tashiro

**Affiliations:** ^1^ Gunma University Heavy Ion Medical Center Maebashi Gunma Japan; ^2^ Department of Radiation Oncology Graduate School of Biomedical and Health Sciences Hiroshima University Minami‐ku Hiroshima Japan; ^3^ Hiroshima High‐Precision Radiotherapy Cancer Center Higashi‐ku Hiroshima Japan; ^4^ RTQM System Inc. Higashi‐ku Hiroshima Japan; ^5^ Gunma University Hospital Maebashi Gunma Japan

**Keywords:** electrometer correction factor, quality assurance, self‐inspection, standard dosimetry

## Abstract

**Background and purpose:**

The standard dosimetry system of medical accelerators in radiotherapy consists of an ionization chamber, an electrometer, and cables. Guidance for TG‐51 reference dosimetry reported that the electrometer correction factor (*P*_elec_) should be checked every few years. Therefore, continuous Pelec measurements have not been reported. The purpose of this study is to measure the Pelec with a charge generator at our institution and to evaluate variations over time. The measurements are compared with calibration data given by an Accredited Dosimetry Calibration Laboratory (ADCL).

**Materials and methods:**

We used four reference‐class electrometers: RT521R (RTQM system/EMF Japan), Model 35040 (FLUKE), RAMTEC Duo (Toyo medic), and UNIDOS‐E (PTW). Each electrometer was connected to the charge generator, and the required charge was applied. The measurement points used were the same as those used for calibration by the ADCL. From the measured charges at each point, the *P*_elec_ was obtained from the slope of the linear regression function. The measurements were repeated over a 3‐month period to evaluate variations over time for each electrometer. Additionally, error budgets for the *P*_elec_ measurements were estimated, and the overall uncertainty was determined.

**Results:**

The measured *P*_elec_ values were 1.0000, 0.9995, 1.0009/0.9999, and 0.9995/0.9998 for RT521R, Model 35040, the low/medium (L/M) ranges of RAMTEC Duo, and the L/M ranges of UNIDOS‐E, respectively. The measured *P*_elec_ values agreed within 0.1% with those given by the ADCL. We found a small drift in the measurements for one electrometer. Additionally, the uncertainty considered was 0.26% for k = 2 (k, coverage factor).

**Conclusion:**

In this study, stable *P*_elec_ values were obtained for four electrometers using a charge generator over a three‐month period. The measured *P*_elec_ values were within the overall uncertainty stated in the electrometer guidelines. However, performing periodic measurements for the *P*_elec_ was able to help in detecting small errors.

## 1 INTRODUCTION

In radiotherapy, the radiation dose is adjusted to provide local control of the tumor and to reduce the occurrence of complications to organs at risk.^1^ Therefore, standard dosimetry is very important to ensure accurate radiation doses. The standard dosimetry system usually consists of an ionization chamber, an electrometer, and connecting cables. The electrometer is one of the most important devices in the standard dosimetry system and collects the charge ionized by radiation in the ionization chamber. Medical electrical equipment—Dosimeters with ionization chambers as used in radiotherapy (IEC 60731) published by the International Electrotechnical Commission (IEC) provides international standard recommendations and defines performance requirements for electrometers.^2^ However, one of the performance requirements IEC 60731 specifies is a 1.6% relative combined uncertainty for ionization chambers and electrometers. This is very large when compared with the relative standard uncertainty of the absorbed‐dose‐to‐water calibration factor. Therefore, the TG‐51 Addendum published by The American Association of Physicists in Medicine (AAPM) points out that the combined relative uncertainty recommended in IEC 60731 is insufficient as a performance requirement.^3^ By following the recommendations in the addendum and performing calibrations with care, a combined relative standard uncertainty in the determination of absorbed dose to water at the reference point can be as low as 1%. More stringent specifications for reference‐class electrometers, including statements on repeatability, long‐term stability, and nonlinearity, were set out. A performance evaluation of electrometers carried out separately from ionization chambers was recommended.^4^, ^5^ Currently, calibration services for electrometers are offered by secondary standard dosimetry laboratories and vendors in various countries, which are traceable to the respective national standard.^3^, ^6^, ^7^, ^8^

Like Accredited Dosimetry Calibration Laboratories (ADCLs) have their calibration directly traceable to the National Institute of Standards and Technology in the United States of America, the Association for Nuclear Technology in Medicine (ANTM) in Japan provides calibration services as a third‐party organization to improve the accuracy and quality control of dosimeters in radiotherapy.^9^ Calibration services have been carried out by combining the calibration of ionization chambers and electrometers using the absorbed dose to water. However, in July 2018, the ANTM started separate calibrations for ionization chambers and electrometers. Consequently, some venders also started providing separate calibration services. In addition, from April 2023, combined calibration of ionization chambers and electrometers will no longer be carried out for some electrometers. Because the calibration of ionization chambers and electrometers is performed separately, electrometers have been assigned an electrometer correction factor (*P*_elec_). The Japanese guidelines for electrometers recommend calibrating the ionization chambers once a year and electrometers once every three years.^10^ In addition, the TG‐51 reference dosimetry states that the reference‐class electrometers should be calibrated when first purchased, following repair, when redundant checks suggest a need, or within intervals not exceeding two years.^11^ Furthermore, Blad et al. recommend regular inspections of the electrometer because it is a very important device of the standard dosimetry system.^5^

Therefore, the purpose of this study is to measure the *P*_elec_ of various electrometers at our institution and to evaluate these measurements. These measurements compared with the calibration data given by the ANTM. We repeated these measurements for three months to evaluate time variations for each electrometer. Additionally, we considered various items to evaluate the uncertainty of the *P*_elec_ measurements.

## 2 MATERIALS AND METHODS

### 2.1 Electrometers

In this study, we used four electrometers of three different types: an integrate mode, a rate mode, and a direct exposure reading mode. We summarized the electrometers used in Table 1. For the electrometers with charge ranges available, measurements were performed for both the Low (L) and Medium (M) ranges.

**TABLE 1:** Electrometers used in this study.

Electrometer	Manufacture	Location	Range	Mode
RT521R	RTQM system/EMF Japan	Japan	–	Integrate
Model 35040	Fluke Electronics Co.	USA	–	Rate
RAMTEC Duo	Toyo Medic	Japan	Low	Direct exposure reading
			Medium	
UNIDOS‐E	PTW Freiburg	Germany	Low	Integrate
			Medium	

RT521R used in this study and EMF521R in ref. 12 are sister products.

### 2.2 Charge generator

The RT521R electrometer has a built‐in electrometer and charge generator, each of which has Triax Connectors (2 stud). The charge generator can be utilized as a current source because the charge is the product of current and time. RT521R has two output terminals, Output‐1 and Output‐2, for the charge generator. The effective currents can generate 20 pA to 2 nA at Output‐1 and 200 pA to 20 nA at Output‐2. The injection time of the charge generator can be set in the range of 0.1 to 1000 s.

The charge generator is calibrated by the manufacturer. The calibration was carried out with a set current and charge measurement; the charge measurement was obtained by measuring the applied set charge for 50 s. Each measurement agreed to the set current within 0.1%. In this study, the value of the current was set as the average current in the calibration. Kinoshita et al. reported that the charge generator can be used to check the accuracy of electrometers.^12^ The calibration data of the charge generator is summarized in Table 2. Each measurement agreed to the set current within 0.1%. The charge was measured with a standard electrometer by applying a set current for 50 s. The average current was obtained by dividing the amount of charge by time and was then compared to the set current. The measured charge was corrected using the electrometer correction factors (*P*_elec_). In this study, we used the average current shown in Table 2.

**TABLE 2:** Calibration data of the charge generator.

Terminal	Set current (pA)	Measured charge (pC)	Average current (pA)	Terminal	Set current (pA)	Measured charge (nC)	Average current (nA)
Output‐1 (+)	+20	1000.220	20.004	Output‐2 (+)	+200	10.00147	200.029
	+200	10 000.030	200.001		+2000	99.99823	1999.965
	+1000	50 000.475	1000.010		+10 000	500.0059	10 000.118
	+2000	100 008.825	2000.177		+20 000	1000.0786	20 001.572
Output‐1 (‐)	−05	−1000.065	−20.001	Output‐2 (‐)	−200	−9.99928	−199.986
	+00	−9999.775	−199.996		−2000	−99.99582	−1999.916
	+05	−49 999.610	−999.992		−10 000	−499.9918	−9999.836
	+10	−100 003.675	−2000.074		−20 000	−1000.0226	−20 000.452

### 2.3 Measurement setup

**FIGURE 1:**
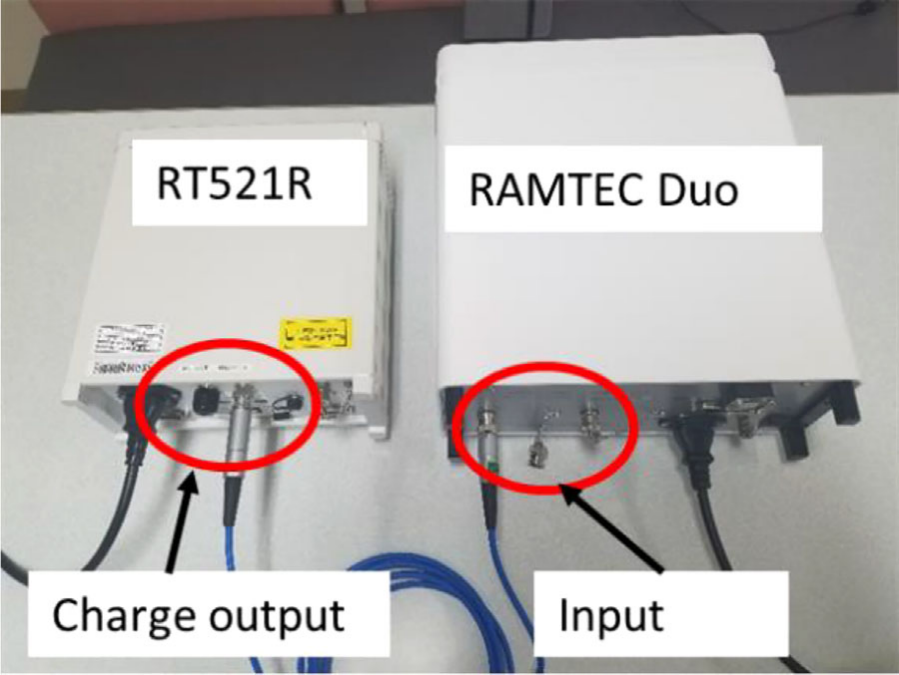
An example of the setup used for measurements. RT521R is connected to RAMTEC Duo with a triaxial cable. In this case, the Output‐2 terminal of RT521R is used.

A 3‐m triaxial cable with Triax Connectors was used to connect the output in RT521R and the input of each electrometer. Figure 1 shows an example of the measurement setup of RAMTEC Duo. RAMTEC Duo is connected to Output‐2 of RT521R. The RAMTEC Duo electrometer measured the direct current applied from the output of the charge generator in RT521R. Since we focused only on electrometers in this study, we did not use any other tools.

The electrometers were used at least two hours after turning on the power, and the zero point was adjusted immediately before measurement. Measurements were taken at room temperature ranging from 22°C to 27°C and relative humidity (RH) ranging from 30% to 70%. The RT521R electrometer can record the internal temperature, current source temperature, and internal RH. In this study, the internal temperature, current source temperature, and internal RH were 34.0 ± 1.0°C, 39.0 ± 1.0°C, and > 10%, respectively.

### 2.4 Measurement of the electrometer correction factor (*P*_elec_)

The Japanese guidelines for electrometers recommend that the measurement points used to determine the *P*_elec_ are based on the points that are widely used in clinical practice.^10^ In this study, the measurement points were set the same as the calibration points used by the ANTM. The measurement points for each electrometer are summarized in Table 3. The current and time used for each calibration point are also summarized. In general, Output‐2 was used for these measurements, while Output‐1 was used when taking measurements with the L range of RAMTEC Duo and UNIDOS‐E.

**TABLE 3:** Summary of the calibration points, current, and time used for each electrometer.

Electrometer	Range					Calibration points						
RT521R	–	Input charge [nC]	+200	+20	+5	−5	−20	−200				
		Current [nA]	+20	+2	+0.2	−0.2	−2	−20				
		Time [s]	10	10	25	25	10	10				
Model 35040	–	Input charge [nC]	+200	+20	+5	−5	−20	−200				
		Current [nA]	+20	+2	+0.2	−0.2	−2	−20				
		Time [s]	10	10	25	25	10	10				
RAMTEC Duo	L	Input charge [nC]	+10	+5	+1	−1	−5	−10				
		Current [nA]	+0.2	+0.2	+0.2	−0.2	−0.2	−0.2				
		Time [s]	50	25	5	5	25	50				
	M	Input charge [nC]	+500	+250	+100	+50	+5	−5	−50	−100	−250	−500
		Current [nA]	+10	+10	+10	+10	+0.2	−0.2	−10	−10	−10	−10
		Time [s]	50	25	10	5	25	25	5	10	25	50
UNIDOS‐E	L	Input charge [nC]	+10	+5	+1	−1	−5	−10				
		Current [nA]	+0.2	+0.2	+0.2	−0.2	−0.2	−0.2				
		Time [s]	50	25	5	5	25	50				
	M	Input charge [nC]	+500	+250	+100	+50	+5	−5	−50	−100	−250	−500
		Current [nA]	+10	+10	+10	+10	+0.2	−0.2	−10	−10	−10	−10
		Time [s]	50	25	10	5	25	25	5	10	25	50

**FIGURE 2:**
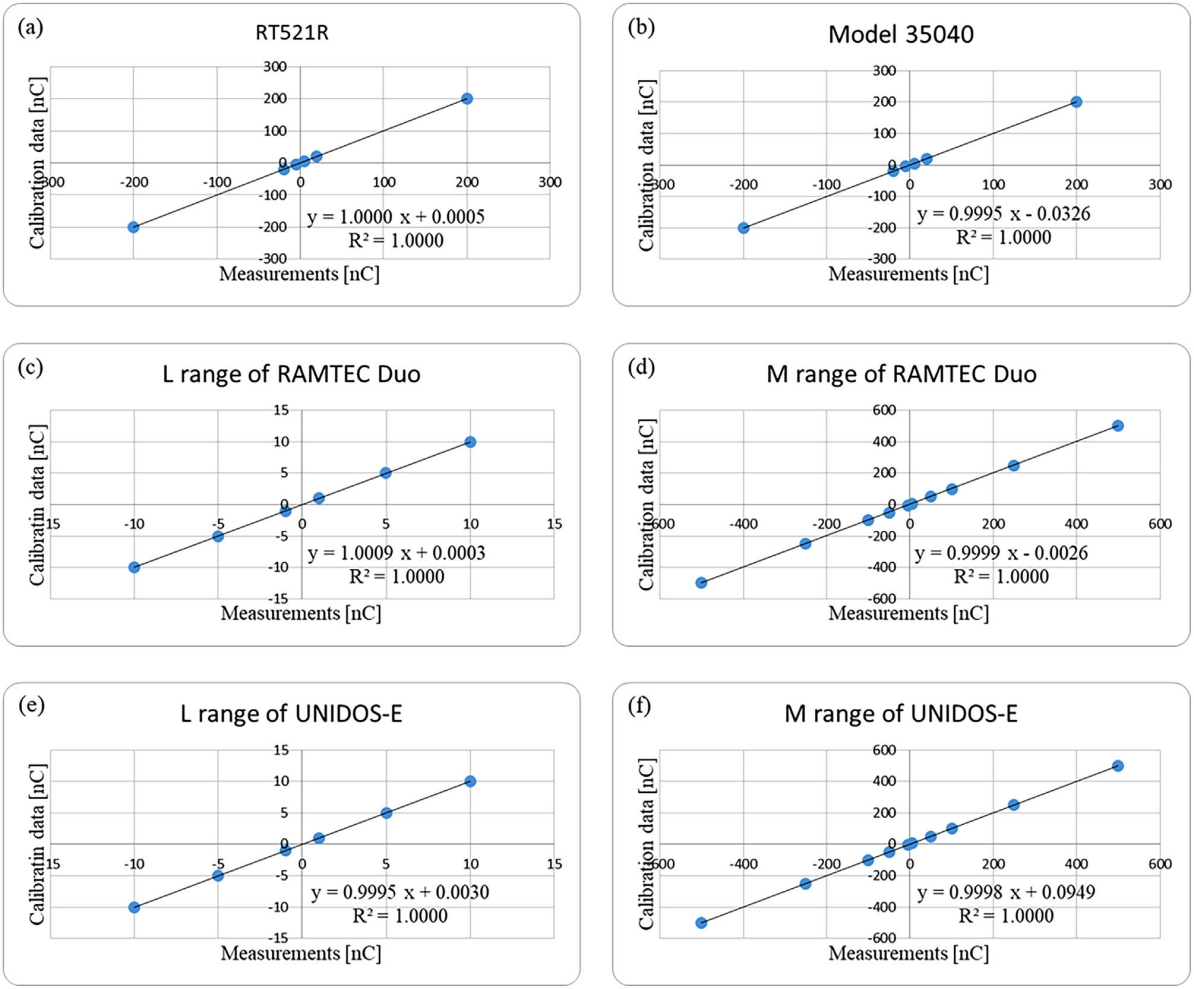
The first measurements of each electrometer and the calibration data. The measurements and calibration data are shown on the horizontal and vertical axis. The measurements for RT521R, Model 35040, the L range of RAMTEC Duo, the M range of RAMTEC Duo, the L range of UNIDOS‐E, and the M range of UNIDOS‐E are shown in parts (a)—(f), respectively.

Plots were created with the measured data on the x‐axis and the calibration data on the y‐axis. The linear function obtained from the linear regression analysis has a slope and y‐intercept, with the slope corresponding to the P_elec_ (Figure 2). The *P*_elec_ values obtained in this study were compared with those provided by the ANTM.

### 2.5 Variation and uncertainty in the *P*_elec_ measurements

The *P*_elec_ measurements were repeated 12 times to evaluate time variations for each electrometer. These 12 measurements were performed over a period of 3 months with intervals of about 1 week.

The *P*_elec_ of each electrometer was obtained using this method. The uncertainty of the measurements was clarified by summarizing uncertainties for various budgets that could have affected the measurements when RT521R was used to apply a current to itself (self‐check). Self‐check refers to connecting the output of the charge generator to the input of the electrometer in RT521R and directly applying a charge to perform a measurement with the electrometer.

## 3 RESULTS

The measurements and the calibration data for each electrometer are shown in Figure 2. These data represent the results of the linear regression from the first‐measurement dataset. The *P*_elec_ values of the four electrometers calibrated by the ANTM were 1.0000, 0.9995, 1.0009, 0.9999, 0.9995, and 0.9998 for RT521R, Model 35040, the L range of RAMTEC Duo, the M range of RAMTEC Duo, the L range of UNIDOS‐E, and the M range of UNIDOS‐E, respectively. All measured *P*_elec_ values, except those for UNIDOS‐E, agreed within 0.1% with the *P*_elec_ values given by the ANTM.

**FIGURE 3:**
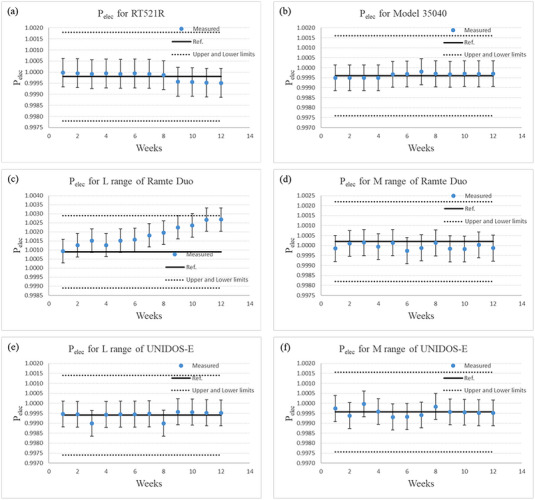
Time variation of the electrometer correction factor (*P*_elec_). Measurement is shown on the horizontal axis; one measurement was performed per week. The *P*_elec_ is shown on the vertical axis. The measurements for RT521R, Model 35040, the L range of RAMTEC Duo, the M range of RAMTEC Duo, the L range of UNIDOS‐E, and the M range of UNIDOS‐E are shown in parts (a)—(f), respectively. Here, the solid line in all figures, except (e) and (f), correspond to the *P*_elec_ obtained from the data provided by the Association for Nuclear Technology in Medicine. The solid line in the figures (e) and (f) corresponds to the average of all measurements. The upper and lower limits shown in dotted lines take into account the 0.2% tolerance for long‐term stability reported in the electrometer guidelines. The error bars indicate the measurement uncertainty of 0.13%.

The measurements taken over a 3‐month period (12 measurements) are shown in Figure 3. All measurements were within the tolerance level for long‐term stability reported in the electrometer guidelines.^10^

Table 4 summarizes the *P*_elec_ values obtained in this study and those obtained from the data provided by the ANTM.

**TABLE 4:** The electrometer correction factor (P_elec_) obtained from the measurement and calibration data.

		Measurements		Calibration data	
Electrometer	Range	*P*_elec_ [nC/rdg]	Standard deviation [%]	*P*_elec_ [nC/rdg]	Uncertainty (k = 2) [%]
RT521R	–	1.0000	±0.02	0.9998	0.16
Model 35040	–	0.9992	±0.01	0.9993	0.20
RAMTEC Duo	L	1.0014	±0.06	1.0009	0.15
	M	0.9999	±0.01	1.0002	0.15
UNIDOS‐E	L	0.9994	±0.02	–	–
	M	0.9996	±0.02	–	–

The error budgets used to calculate the uncertainty of these measurements are summarized in Table 5. These error budgets are considered for the case when RT521R is inspected using the RT521R charge generator (self‐check). The data in the first and second rows correspond to the electrometer uncertainty data provided by the ANTM (type B uncertainty) and the standard deviation of repeated measurements (type A uncertainty), respectively. The data in the remaining rows are obtained from the manufacturer's catalog (type B uncertainty). Since RT521R uses an integrate mode, zero shift and charge leakage were not considered. The overall uncertainty was determined by combining all uncertainties using the root mean square. The combined uncertainty was 0.13% for k = 1 (k, coverage factor). This value is similar to one of the combined uncertainties reported by Kinoshita et al. (1.14%, k = 1).^12^

**TABLE 5:** Error budgets for this study.

Budgets of uncertainty	Uncertainty (%, k = 1)	Comments
Charge measurement of electrometers	0.08	Provided by the ANTM*‐accredited facility
Repeatability (measurements)	0.01	Obtained from measured data
Time linearity of output charge	0.01	Obtained from catalog
Uncertainty of zero drift	0.1	Obtained from catalog
Temperature coefficient of zero drift (/°C)	0.015	Obtained from catalog
Temperature coefficient of output current (/°C)	0.003	Obtained from catalog
Combined uncertainty (k = 1)	0.13	Root Mean Square
	* Association for Nuclear Technology in Medicine	

## 4 DISCUSSION

The *P*_elec_ measurements for various electrometers using the charge generator could be performed with sufficient accuracy at our institution. We discuss below the measurement drift, the uncertainties of this method, and the limitations of this study.

The measurements performed over a period of three months showed that the *P*_elec_ values for all electrometers agreed within 0.2% with those given by the ANTM, with 0.2% being the tolerance for the long‐term stability recommended by the electrometer guidelines.^9^, ^10^ However, we found a drift in the measurements for the L range of RAMTEC Duo (Figure 3). Therefore, we checked items such as the zero shift, zero drift, and charge leakage for the L range of RAMTEC Duo. All items were acceptable within the catalog values. Subsequently, we requested the calibration data of RAMTEC Duo be measured again at the ANTM about six months after the first calibration. The calibrated measurements were 1.0026 for the L range and 1.0005 for the M range. The 12^th^ measurements were 1.0027 for the L range and 0.9999 for the M range agreeing within 0.1% with the calibrated data. This shows that the *P*_elec_ measured using RT521R was correct. Then, we considered the possible reasons causing this variation. First, this electrometer uses an integrate mode, thus requiring appropriate control of environmental conditions, such as temperature and humidity. In particular, the charge variation is relatively large in the L range, where high accuracy is required to handle small signals. All electrometers were placed at room temperature, but it may have been necessary to store them in a desiccator, especially the electrometers with integrate mode. Second, there is the possibility of initial variations after first purchase of the electrometers. In case of RAMTEC Duo, the *P*_elec_ measurements started immediately after purchase, while in case of the other electrometers, the *P*_elec_ measurements were carried out at least one year after purchase. It is possible that the electrometers took time to reach performance stability. After the second calibration of RAMTEC Duo, we continued taking *P*_elec_ measurements at our institution. The results were similar to those of the 12^th^ measurements. The *P*_elec_ for the L range of RAMTEC Duo was 1.0029 for the 13^th^ measurement (one month after the second calibration) and 1.0026 for the 14^th^ measurement (two months after the second calibration). Initial variations can occur in any electrometer. Although we cannot identify a clear reason, we were able to detect the drift of the *P*_elec_ by repeating measurements. The presence of absolute deviations in the *P*_elec_ values, like the initial variations observed in our study, will directly affect the dose control of medical linear accelerators (LINACs) as systematic errors if they remain undetected. Therefore, variations of the *P*_elec_ should be detected and eliminated. Performing periodic measurements for the *P*_elec_ can help in detecting errors that cannot be detected through measurements of the zero shift, zero drift, or charge leakage, which recommended for electrometers by IEC 30731 and the Institute of Physics and Engineering in Medicine (IPEM).^2^, ^4^

The uncertainty estimated in this study was 0.26% for k = 2. Some components of the uncertainty are based on catalog values, which are larger than the actual uncertainties. The combined uncertainty of the items listed in the guidelines of TG‐51 reference dosimetry was 0.35%.^10^ Therefore, it is considered that the *P*_elec_ can be obtained within the accuracy indicated in the guidelines. However, the uncertainty for the calibration data of the electrometers used in our study were less than 0.2% for k = 2 as given by the ANTM. Thus, it is not recommended to determine the *P*_elec_ using the charge generator of the RT521R electrometer. In addition, cross‐calibration, which compares two electrometer readings using the same ionization chamber and the same irradiation to obtain the *P*_elec_, is performed at many facilities. The LINAC output and setup errors, and variations based on the type of ionization chambers are included in cross‐calibration,^13^, ^14^, ^15^ so it is difficult to detect an error of about 0.1%. Therefore, the inspection of electrometers using a charge generator could be an effective quality management for medical facilities.

The limitation of this study is the short period of three months used to carry out the measurements. We believe that a longer period should be considered so as to more accurately check the electrometers. A future study will consider a longer period and thus, a larger dataset.

## 5 CONCLUSION

In this study, we could obtain *P*_elec_ values using RT521R as a charge generator at our institution. We showed that the *P*_elec_ can be obtained stably over a 3‐month period. In addition, the examination of the uncertainty suggested that the measured P_elec_ values were within the acceptable range of the tolerance level specified by the electrometer guidelines.

## AUTHOR CONTRIBUTIONS

The authors confirm contribution to the paper as follows: study conception and design: Motohiro Kawashima, and Shuichi Ozawa; data collection: Motohiro Kawashima, Hiroki Okada, Hiromitsu Higuchi, and Yoshihiko Hoshino; analysis and interpretation of results: Motohiro Kawashima, Maria Varnava, and Shuichi Ozawa; draft manuscript preparation: Motohiro Kawashima, Maria Varnava, Shuichi Ozawa, and Mutsumi Tashiro. All authors reviewed the results and approved the final version of the manuscript.

## CONFLICT OF INTEREST STATEMENT

Motohiro Kawashima reports financial support was provided by RTQM system Inc. Shuichi Ozawa reports a relationship with RTQM system Inc. that includes board membership and equity or stocks.
